# Pyogenic Liver Abscess: A Case of a Fussy Bug Seeking Unlikely Host

**DOI:** 10.7759/cureus.42263

**Published:** 2023-07-21

**Authors:** Amir R Reihani, Fernanda Ponce, Roman Spivak

**Affiliations:** 1 Medical Education, Griffin Hospital/Yale-Affiliated Hospital, Derby, USA; 2 Pulmonary and Critical Care Medicine, Eisenhower Hospital, Rancho Mirage, USA; 3 Pulmonary and Critical Care Medicine, Griffin Hospital/Yale-Affiliated Hospital, Derby, USA

**Keywords:** amebic liver abscesses, covid-19 pneumonia, complications, immunocompetent, fusobacterium liver abscess

## Abstract

Pyogenic liver abscesses (PLAs) represent a rare yet life-threatening condition, and *Fusobacterium* species are an atypical etiology typically associated with immunocompromised patients or those with recent dental procedures or oro-gingival disease. Nonetheless, it is crucial to maintain a high level of suspicion for *Fusobacterium* infection in all cases, including immunocompetent individuals without known risk factors. In this case report, we present the clinical scenario of a young male with a history of COVID-19 pneumonia who exhibited subacute fever, abdominal pain, and pleuritic chest pain, leading to sepsis attributed to intraabdominal pathology. Subsequent imaging confirmed the presence of possible liver abscesses, prompting interventional radiology-guided drainage. Cultures obtained from the drained abscesses yielded *Fusobacterium* species, and significant clinical improvement was observed following the initiation of appropriate antibiotic therapy. This case report underscores the potential for disseminated *Fusobacterium* infections to occur in immunocompetent individuals without a history of oropharyngeal disease, highlighting the importance of early diagnosis and management to mitigate mortality risk.

## Introduction

Pyogenic liver abscesses (PLAs) are a rare life-threatening disease with an estimated incidence of 2.3 cases per 100,000 population [1,2,3]. While they are typically polymicrobial, monomicrobial PLAs do occur, among which *Fusobacterium* species are a particularly unusual cause. In a review that was published by Nagpal et al. in 2015, it was reported that PLAs due to these rare oropharyngeal pathogens are normally seen in immunocompromised individuals with a history of recent dental work/instrumentation or oro-gingival disease [2]. Given the potential mortality associated with PLAs however, high suspicion for an occult source of infection such as *Fusobacterium* should always be maintained, even in immunocompetent individuals without known risk factors.

## Case presentation

**Figure 1:**
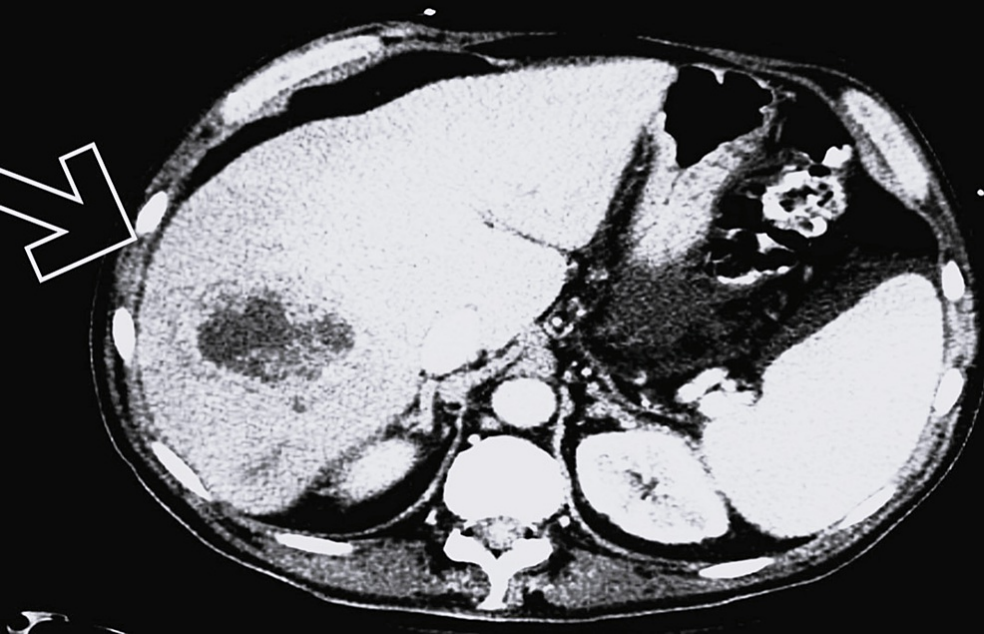
Abdominal computed tomography imaging revealing hepatic abscess, prior to drainage and antibiotics

A 26-year-old male with a past medical history significant for COVID-19 pneumonia three months prior to admission presented with fever, chills, nausea, vomiting, diarrhea, right upper quadrant abdominal pain, dyspnea, and pleuritic chest pain that progressed gradually over three weeks. He was septic on presentation with initial vital signs including a heart rate of 112 beats per minute, a respiratory rate of 35 breaths per minute, and severe hypoxia with an oxygen saturation of 86% on room air, requiring 4.5 liters of supplemental oxygen to achieve saturation above 94%. The physical examination was otherwise significant for right upper quadrant pain, though no rebound tenderness was noted. Laboratory investigations revealed leukocytosis, with a WBC count of 14,000/mm³ and elevated granulocytes, but no bandemia. Procalcitonin levels were mildly elevated, along with lactic acid levels. Notably, liver function tests showed a twofold elevation in liver enzymes, accompanied by increased levels of inflammatory markers, including C-reactive protein (CRP) moderately elevated at 35 mg/L, lactate dehydrogenase (LDH) increased at 550 U/L, and D-Dimer at a level of 700 ng/mL. Imaging revealed a large liver abscess measuring 3.9 cm × 4.4 cm × 3.5 cm (Figure 1). 

The 26-year-old male was admitted to the intensive care unit with a preliminary diagnosis of sepsis secondary to presumed amebic liver abscesses and started on empiric ceftriaxone and metronidazole. The abscesses were drained under CT guidance with subsequent percutaneous drain placement. Blood cultures remained negative, but aspirate cultures grew *Fusobacterium* species, and antibiotics were consequently switched to ampicillin-sulbactam. He denied recent dental procedures and appeared to have good oral hygiene. His hospital course was complicated by the following: portal vein thrombosis, recurrent pleural effusions requiring multiple thoracenteses, and biliary fistula formation between the abscess cavity and the intrahepatic bile ducts requiring endoscopic retrograde cholangiopancreatography (ERCP) sphincterotomy with stent placement. Symptoms improved gradually after two weeks of IV antibiotics, specifically Unasyn, administered at a standard dose of 3 g every six hours. Following this, he was discharged with a six-week course of the oral 𝛃-lactam antibiotic, amoxicillin-clavulanate, at a dosage of 500 mg/125 mg three times a day. Repeat CT of the abdomen 10 weeks after presentation showed complete resolution of the hepatic abscesses.

## Discussion

*Fusobacterium* species, characterized as gram-negative anaerobic bacilli, have been implicated in a wide range of human ailments, spanning from localized oral infections to severe systemic conditions such as Lemierre's syndrome and PLAs [1]. PLAs caused by *Fusobacterium*, however, remain a rarity with only a minority of reported cases indicating it as the causative organism [3,4].

Our study presents an unusual case involving an otherwise healthy individual who developed complications of Fusobacterium infection, devoid of any indication of *Fusobacterium *bacteremia or oropharyngeal disease [2]. This finding, enhancing our understanding of *Fusobacterium* pathogenesis, implies that disseminated *Fusobacterium* infections may occur in immunocompetent individuals lacking evidence of oropharyngeal disease [3,5].

Despite a well-established association of *Fusobacterium *infections with immunocompromised conditions and oro-gingival diseases/instrumentation [3], our case, alongside reports like that of Kuppalli et al., in 2012, serves to underline the ability of *Fusobacterium* to precipitate severe disease in healthy individuals devoid of apparent predisposing factors [6].

Additionally, recent studies have identified unexpected manifestations of *Fusobacterium* infections, like in the report by Riordan T., in 2007, where *Fusobacterium necrophorum* was discovered as the causative agent of vertebral osteomyelitis in an adolescent [7]. Such atypical presentations underscore the unpredictable nature of *Fusobacterium* infections and highlight the importance of including this pathogen in differential diagnosis across a wide array of infectious conditions. Furthermore, the role of the patient's recent COVID-19 infection in the presentation remains uncertain, presenting an opportunity for further research to explore a possible association between anaerobic infections and COVID-19 [4].

Given the potential for severe complications and elevated mortality rates linked with PLAs, maintaining high suspicion of *Fusobacterium *infection is crucial, even in the absence of known risk factors [8]. Altemeier et al., in 1973, noted that the liver is primarily where intraabdominal abscesses occur, yet it is unclear why *Fusobacterium nucleatum* seems to prefer the liver over other internal organs [9]. This could be attributable to the liver's unique dual blood supply derived from both the portal and systemic circulations. Alternatively, liver abscesses during *Fusobacterium bacteremia* might signify an early disease stage and, if left untreated, could eventually affect other organs [9]. There may also exist unexplored factors and specific interactions between the organism (*Fusobacterium nucleatum*) and the liver.

Prompt detection and treatment of PLA cases are essential due to the high mortality risk in untreated patients [10]. Risk factors escalating mortality include an anaerobic infection, abscesses larger than 5 cm, and the necessity for open surgical drainage, as per the findings of Yang et al. (2017), Lok et al. (2008), and Chen et al. (2012) [10,11,12]. Typically, treatment necessitates abscess drainage coupled with the administration of appropriate antibiotics. Ideally, cultures should be collected before initiating antibiotic therapy to maximize treatment efficacy [13].

## Conclusions

Considering the potential mortality associated with pyogenic liver abscess, it is crucial to uphold a heightened level of suspicion for an occult source of infection, such as *Fusobacterium*. This remains true even in immunocompetent individuals without known risk factors.
